# Characteristics and hemolysis analysis of centrifugal blood pumps under different speed modulations

**DOI:** 10.3389/fphys.2025.1575971

**Published:** 2025-04-25

**Authors:** Chengxuan Su, Donghai Jin, Guangmao Liu, Shulei Li, Xingmin Gui

**Affiliations:** ^1^ School of Energy and Power Engineering, Beihang University, Beijing, China; ^2^ Fuwai Hospital State Key Laboratory of Cardiovascular Disease, Beijing, China

**Keywords:** centrifugal blood pump, hemolysis, various speed modulation, computational fluid dynamics, flow field

## Abstract

**Introduction:**

Constant-speed methods are widely applied and studied in rotary blood pumps. However, various speed modulation which have been used in commercial blood pump lacks validation of the ventricular assist capability and hemolysis potential.

**Methods:**

This study investigates the hydrodynamic performance and hemolysis of a rotary ventricular assist device under sinusoidal speed modulation, focusing on the combined effects of phase, baseline speed, and speed fluctuation amplitude.

**Results:**

Computational fluid dynamics (CFD) coupled with a dynamic cardiovascular model revealed that counter-phase modulation reduces hemolysis index (HI) fluctuations compared to in-phase conditions, while higher baseline speeds increase time-averaged HI due to prolonged exposure to non-physiological shear stress. Larger amplitudes expand the operational range but exacerbate HI variability.

**Discussion:**

These findings demonstrate that phase synchronization critically balances pulsatility and hemocompatibility, providing actionable insights for adaptive speed control strategies in clinical practice.

## 1 Introduction

Heart failure is a critical global health issue with increasing prevalence, highlighting the urgent need for effective treatments. Mechanical circulatory support (MCS) devices, such as ventricular assist devices (VADs), have become essential in managing heart failure, especially as a bridge to transplantation or as destination therapy for patients ineligible for heart transplants (MacIver and Ross, 2012; Gustafsson and Rogers, 2017; Frankfurter et al., 2020; Han et al., 2022).

While most commercially available continuous-flow VADs operate at a constant rotational speed, this fixed-speed approach has notable limitations (Slaughter et al., 2010). The physiological demands on the cardiovascular system vary significantly with changes in physical activity, posture, and emotional states. A constant-speed pump cannot adequately adapt to these dynamic conditions, potentially leading to suboptimal hemodynamic support and increased risks of adverse events, such as thrombus formation and hemolysis (Alkan et al., 2007).

The human cardiovascular system inherently operates in a pulsatile manner, driven by the rhythmic contraction of the heart. This pulsatility is not merely a mechanical phenomenon but is tightly coupled to physiological functions such as endothelial shear stress regulation, nitric oxide release, and microvascular perfusion. Constant-speed VADs, by suppressing natural pulsatility, may disrupt these processes, leading to endothelial dysfunction and impaired organ perfusion. Our study investigates how variable-speed modulation can restore pulsatile flow characteristics, thereby aligning mechanical support with the physiological requirements of dynamic shear stress profiles and vascular adaptation. Variable-speed blood pumps offer a promising solution by dynamically adjusting the pump speed to align better with the patient’s physiological needs (Ising et al., 2015). This adaptability can enhance the hemodynamic performance, reduce complications, and improve the patient’s quality of life (Choi et al., 2001; Liao et al., 2018). Both HVAD and HeartMate 3 devices have adopted their own speed modulation methods, called Lavage cycle and artificial pulse, to create artificial pulses, thereby combining the benefits of continuous flow with pulsatile flow characteristics, and preliminary clinical trials have been conducted (Netuka et al., 2015; Bourque et al., 2006; 2016). As new speed modulation types may emerge and these variable speed modulations can be realized in practical application (Soucy et al., 2015; Harada et al., 2021), understanding hemolytic performance under varying conditions has become crucial.

At present, some studies have reported the variable speed issues of blood pumps. Wang et al. (2019) studied the effects of pulsatile waveform speed and sinusoidal waveform speed on hemolysis compared to steady speed blood pumps. Huang et al. (2023) investigated the effects of variable speed blood pump waveforms on the hemolytic performance of blood pumps, concluding that the effects of triangular waves, sinusoidal waves, and square waves on the hemolytic performance of blood pumps are not significant. Huo et al. (2024) studied the effects of blood pumps under constant and pulsatile speed modulation on local hemodynamic parameters of the aorta, indicating that pulsatile speed increases the blood flow fluctuation in the aorta. However, the dynamic interactions between speed modulation parameters (phase, baseline speed, and amplitude) and physiological cardiac cycles require further research. Furthermore, as blood pumps function as fluid pumps, their operational states and characteristic curves (e.g., pressure-flow hysteresis) are critical evaluation metrics in hydraulic engineering. Therefore, when assessing speed modulation strategies, it is essential not only to consider hemolytic properties but also to analyze the pump’s working behavior under varying cardiac states, such as ventricular pressure fluctuations and vascular resistance changes.

This research focuses on centrifugal blood pumps in extracorporeal systems, highlighting their potential for variable speed control to enhance patient outcomes. The study aims to explore the relationship between different baseline speeds of the blood pump, speed fluctuation amplitudes, and the phase differences with the left ventricular pressure waveform using numerical simulations. Additionally, the study aims to explore the connections between hemolysis, flow distributions, and hydrodynamic characteristics of the blood pump under various variable speed waveform modulations ultimately aiming to advance MCS device effectiveness.

## 2 Methodology

### 2.1 Geometry

**FIGURE 1:**
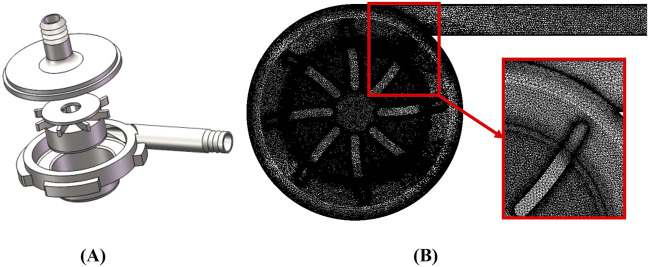
**(A)** The geometry of the centrifugal pump; **(B)** The mesh details of the centrifugal pump.

The object of this research is a self-developed, magnetically levitated centrifugal blood pump suitable for various external MCS systems. As shown in Figure 1A, the pump housing and impeller are made of transparent polycarbonate. The inlet and outlet ports are designed as pagoda joints, featuring 90° angles. The blood pump has a closed impeller with eight blades and a central hole. The overall height of the pump is 56.5 mm, and the duct diameter is 70 mm. The inlet and outlet diameters are 9.4 mm and 9.0 mm, respectively.

The blood pump’s impeller is suspended both axially and radially during rotation, with rotor support achieved by a radial magnetic suspension bearing. A magnet is located beneath the impeller. The blood pump can provide a flow range of 1–7 L/min, with an impeller speed range of 1,000–5,000 rpm. Under design conditions, the pump achieves a flow rate of 4 L/min and a pressure difference of 104 mmHg.

### 2.2 CFD setup and experiment validation

Commercial CFD software, CFX 2020R1 (ANSYS Inc., Canonsburg, PA), was employed for the numerical simulations. The hematological properties of human blood were simulated using an incompressible fluid with a density of 1.055 kg/m^3^ and a dynamic viscosity of $3.5\times 10^{-3}\;Pa\cdot s$ (Ghadimi et al., 2019; Liu et al., 2019). The SST-ω turbulence model was chosen for the simulations (Thamsen et al., 2015; Granegger et al., 2020; Li et al., 2023b). Convergence criteria were set to a root mean square error (RMSE) of less than $1\times 10^{-4}$ . Over 99.99% of the mesh elements met the following criteria: maximum element volume ratio <170°, aspect ratio <100, skewness <0.54, and orthogonal quality >0.45. The y-plus value was kept below one to satisfy the requirements of the SST turbulence model. We conducted steady-state simulation calculations with the mesh elements ranging from 1.82 million to 14.25 million, and verified mesh independence at the operating point with a rotational speed of 2000 rpm, a flow rate of 4 L/min, and an inlet static pressure of 10 mmHg. When the number of meshes reaches 11.27 million, the change in the hemolysis index with the number of mesh elements is no longer significant, and the change in the hemolysis index obtained from the simulation compared to the 14.25 million mesh number is less than 0.52%. Therefore, we chose the 11.27 million mesh blood pump mesh for further research and analysis. The generation details are shown in Figure 1B with no negative volume elements.

The initial condition of transient simulations was derived from a simulation at steady-state operation. During the steady simulations, a static pressure of 10 mmHg was defined at the inlet, and mass flow rate conditions were specified at the outlet. The transient simulation results show that the difference of the pressure rise and hemolysis index between the third cardiac cycle and the second cardiac cycle is less than 1%. Therefore, the results in the third cardiac cycle are chosen as the final calculated results after initializing for two cycles (1.2 s) under the condition of a heart rate of 100 beats per minute (bpm). The time step for all CFD simulations was set to 0.001 s.

**FIGURE 2:**
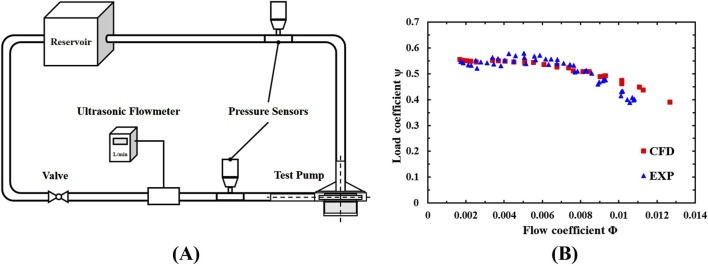
**(A)** The mock circulatory loop rig; **(B)** Normalized flow rate-pressure rise characteristic curve.

A mock circulatory loop rig was established for the validation and numerical results as shown in Figure 2A. The rig includes a reservoir, PVC pipes, two dynamic pressure sensors, an ultrasonic flowmeter (10PXL probe, TS410 m, Transonic Systems, United States), a flow control valve, and the centrifugal blood pump. The mock loop is filled with a glycerol and deionized water mixture (60/40 mass percentage) to simulate the blood’s density and dynamic viscosity of $3.5\times 10^{-3}\;Pa\cdot s$ . Flow rates ranging from 1 to 7 L/min (in 0.5 L/min intervals) were achieved by adjusting the flow control valve, while the blood pump operated at rotational speeds of 1,600, 1800, 2000, 2,200, and 2,400 rpm in sequence.

The experiment data were normalized as the flow coefficient ϕ and load coefficient ψ according to Equations 1, 2. This normalization converges the pump flow-pressure characteristics of different pump speeds into a specific curve.

$$\phi =\frac{G}{\rho u_{2}d_{2}^{2}}$$ (1)

$$\psi =\frac{\Delta P}{\rho n^{2}d_{2}^{2}}$$ (2)

Where $u_{2}$ represents the linear velocity at the trailing edge of impeller, and can be obtained by $u_{2}=\frac{2\pi n}{60}\cdot \frac{d_{2}}{2}$ . $d_{2}$ is the diameter of the trailing edge of impeller, and n represents the pump rotational speed.

The hydraulic characteristics of the blood pump under different rotational speeds were obtained, with a maximum pressure rise deviation of 1% between experimental and CFD results. Figure 2B compares the normalized experimental results and CFD results, showing good consistency and confirming the reliability and accuracy of the numerical method.

### 2.3 Hemolysis calculation in CFD

Scalar shear stress is a critical factor in hemolysis calculation. The shear stress experienced by red blood cells and the residence time within a certain region are the main factors causing hemolysis, so the current evaluation of hemolysis in blood usually adopts the power law model proposed by Gierschpen as Equation 3 (Giersiepen et al., 1990):

$$HI=\frac{∆Hb}{Hb}\left(\%\right)=Ct^{\alpha }\tau^{\beta }$$ (3)

Where Hb represents the total hemoglobin concentration, ∆Hb is the released hemoglobin concentration, t is the exposure time, τ stands for scalar shear stress.

The constant parameters of the original model was obtained from the fitting measurement data discussed in the reference (Wurzinger et al., 1985). Heuser and Opitz proposed a set of parameters from experiments on pig blood (Heuser and Opitz, 1980), which have been widely used and validated (Li et al., 2023b; Su et al., 2024). Zhang et al. proposed another set of parameters from hemolysis experiments on ovine blood (Zhang et al., 2011) and have been utilized in several studies (He et al., 2021; Han et al., 2024). The constants C , α , β used in this study are from Heuser’s model: $C=1.8\times 10^{-8}$ , α=0.765 , β=1.991 . The scalar shear stress τ is determined using Equation 4 (Giersiepen et al., 1990):

$$\tau ={\left[\frac{1}{6}\sum {\left(\tau_{ii}-\tau_{ij}\right)}^{2}+\sum \tau_{ij}^{2}\right]}^{1/2}$$ (4)

Hemolysis was calculated within the CFD simulations to assess the blood damage potential of the pump under different operational conditions. The Euler method was used to calculate the hemolysis distribution by solving Equation 5 (Bludszuweit, 1995; Yokoyama et al., 2010):

$$\frac{d\left({HI}^{1/\alpha }\right)}{dt}=\frac{\partial \left({HI}^{1/\alpha }\right)}{\partial t}+v\cdot \nabla {HI}^{1/\alpha }=C^{1/\alpha }\tau^{\beta /\alpha }$$ (5)

Where $C^{1/\alpha }\tau^{\beta /\alpha }$ is the source term, and was set as 0 at the inlet to simulate the absence of hemolysis at the entrance and was added into each subdomain in the pump model. Gauss’s flux theorem is applied to calculate the average linear damage of blood as Equations 6, 7, assuming a uniform distribution of damage throughout the blood pump.

$$\int_{V}\left(v\cdot \nabla {HI}^{1/\alpha }\right)dV=\int_{V}\left(C^{1/\alpha }\tau^{\beta /\alpha }\right)dV=\int_{S}\left(v\cdot \vec{n}\right){HI}^{1/\alpha }dS=\bar{{HI}^{1/\alpha }}\cdot Q_{out}$$ (6)

$$HI={\left(\bar{{HI}^{1/\alpha }}\right)}^{\alpha }={\left(\frac{1}{Q_{out}}\int_{V}\left(C^{1/\alpha }\tau^{\beta /\alpha }\right)dV\right)}^{\alpha }$$ (7)

Where dS is the cross-section element at the outlet, $Q_{out}$ is the outlet flow rate of the blood pump. The mass-weighted average value of HI at the pump outlet is regarded as the final hemolysis performance.

### 2.4 Hemodynamic conditions setup

**FIGURE 3:**
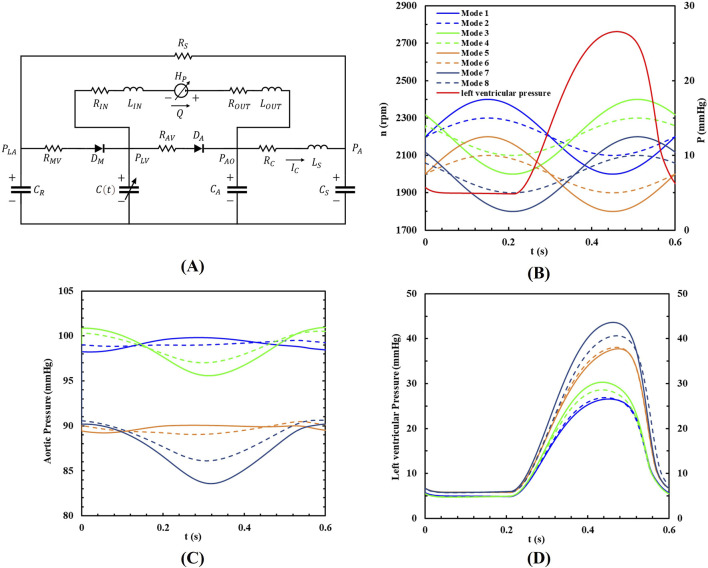
**(A)** The LPM of the coupled system; **(B)** The eight pump speed modulations, **(C)** the aortic pressure and **(D)** the left ventricular pressure within a cardiac cycle.

The lumped parameter model (LPM) of the cardiovascular system used in this study is based on the electrical analogy method proposed by Simaan et al. (2009), Li et al. (2023a), and was used to evaluate the coupled working state of blood pumps and the cardiovascular system. Figure 3A illustrates the complete model of the cardiovascular system and its connection to the blood pump.

The left ventricular assist device (LVAD) is connected from the left ventricle to the aorta, with catheter inertia and resistance included in the model. The cardiovascular model comprises the left heart circulation and other systemic pathways. The heartbeat is modeled as a time-varying nonlinear elastic system, where the elasticity values represent the heart’s contractility. These values are adjusted to simulate different heart failure conditions. Valves are represented by ideal diodes to prevent blood backflow. The systemic arterial system is modeled using a four-element Windkessel model, which simulates resistance, compliance, and inertia (Wang et al., 2019). The venous system, acting as the primary blood reservoir, exhibits concentrated compliance under the influence of resistance. The specific parameters are provided in Table 1.

**TABLE 1:** The LPM parameters.

Resistance ( mmHg·s/mL )		Compliance ( mL/mmHg )		Inertia ( $mmHg\cdot s^{2}/\text{mL}$ )		Elasticity ( mmHg·s/mL )	
$R_{S}$	1.0000	$C\left(t\right)$	$1/E\left(t\right)$	$L_{S}$	0.0005	$E_{\max}$	1.05
$R_{MV}$	0.0050	$C_{R}$	4.4000	$L_{IN}$	0.0001	$E_{\min}$	0.1
$R_{AV}$	0.0010	$C_{S}$	1.3300	$L_{p}$	−0.0035		
$R_{C}$	0.03968	$C_{A}$	0.0800	$L_{OUT}$	0.0099		
$R_{IN}$	0.0001			L	0.01		
$R_{OUT}$	0.0065						

Symbols: P , pressure (mmHg); I , blood flow rate, L , inertia, R , resistance, C , compliance, D , unidirectional flow of the valve (diode function), $C\left(t\right)$ , Left ventricular compliance, $H_{P}$ , pressure rise generated by LVAD, Q , flow rate across LVAD.

Subscripts: AO , aorta, A , arteries, AV , aortic valve, S , systemic vascularity, LA , left atrium, MV , mitral valve, LV , left ventricular, IN , inflow cannula of the LVAD, OUT , outflow cannula of the LVAD.

The dynamic hydraulic characteristic of the blood pump is governed by an ordinary differential equation together with the cardiovascular system as Equation 8. Specifically, the pressure rise of the blood pump depends on the flow rate and rotational speed, which were determined by regression analysis of the experimentally measured pressure head and flow rate. Besides, it also includes a term to account for the inertia effect of the blood pump (Tang et al., 2012):

$$P=a_{0}+a_{1}Q+a_{2}Q^{2}+a_{3}n+a_{4}n^{2}+L\frac{dQ}{dt}$$ (8)

Where P is the pump pressure head, Q is the flow rate, n is the pump rotational speed, t is the time, L represents the inertia effect. The coefficients $a_{x}$ were calculated from the steady results from the experiments: $a_{0}=-0.199$ , $a_{1}=0.6676$ , $a_{2}=-0.0088$ , $a_{3}=-0.0208$ , $a_{4}=2.94\times 10^{-5}$ .

To analyze various operational conditions, the study simulated eight different support modes based on two types of sinusoidal waveforms. One waveform was in-phase with the left ventricle, meaning the peak left ventricular pressure coincided with the highest pump speed. The other waveform was out-of-phase, meaning the peak left ventricular pressure coincided with the lowest pump speed. Baseline pump speeds were set at 2,200 rpm and 2,000 rpm, with speed fluctuations of 200 rpm and 100 rpm, respectively. The pump’s rotational speed followed a sine waveform, and its phase position relative to the left ventricular pressure waveform is shown in Figure 3B. In this study, the heart rate is set to a constant 100 beats per minute to explore the impact of different rotational speed modulation on blood pump assistance. Therefore, the period of the waveforms was set to 0.6s, which has the same period as the heartbeat. All the speed setups are shown in Table 2.

**TABLE 2:** Variable pump speed modulations.

Speed setting mode name	Baseline speed ( $n_{0}$ , rpm)	Speed fluctuations (A, rpm)	Phase condition
Mode 1	2,200	200	counter-phase
Mode 2	2,200	100	counter-phase
Mode 3	2,200	200	in-phase
Mode 4	2,200	100	in-phase
Mode 5	2000	200	counter-phase
Mode 6	2000	100	counter-phase
Mode 7	2000	200	in-phase
Mode 8	2000	100	in-phase

The input parameters of CFD transient simulations were calculated by the integrated numerical model that combines the hysteresis model with the cardiovascular system under the heart failure conditions, and the inlet pressure was set as left ventricular pressure $P_{LV}\left(t\right)$ , and the outlet pressure was set as the sum of $P_{LV}\left(t\right)$ and the pump pressure $P_{P}\left(t\right)$ , where $P_{LV}\left(t\right)$ and $P_{P}\left(t\right)$ are pressure functions of time, calculated by the integrated numerical model that combines the hysteresis model with the cardiovascular system under the heart failure conditions.

## 3 Results

### 3.1 Characteristics under variable speed conditions

**FIGURE 4:**
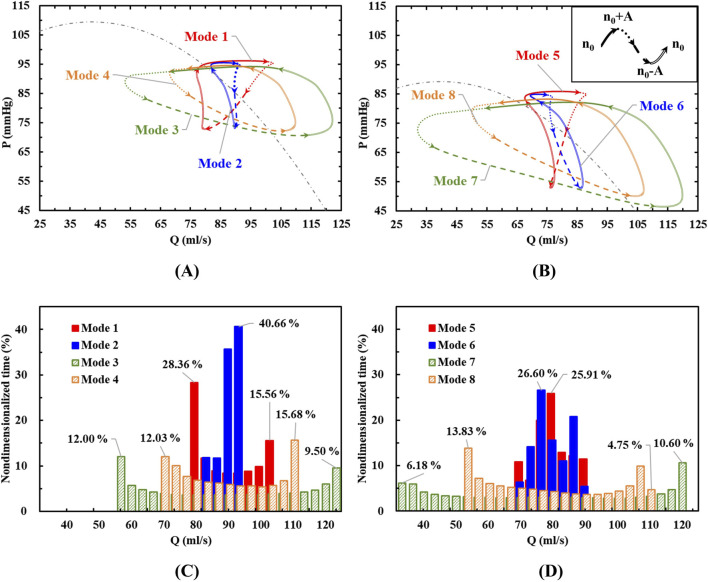
**(A, B)** The characteristic loops in eight different modes divided into four stages; **(C, D)** The flow rate distributions during a cardiac cycle.

The simulated flow rate-pressure rise characteristic loops during one heart beat cycle are shown in Figure 4A and (B) for the eight different speed modulation conditions. As indicated in Figures 3C, D, the aortic pressure is always higher than the left ventricular pressure, ensuring the aortic valve remains closed. For comparison, each characteristic loop is divided into four parts: n0∼n0+A, n0+A ∼ n0, n0∼n0-A, n0-A ∼ n0, where n0 is the baseline speed and A is the speed fluctuation in each speed modes. To analyze the flow distribution characteristics of blood pumps under different speed modulations, the time spent by flows in different ranges within a cardiac cycle was statistically evaluated as shown in Figures 4C, D. The nondimensionalized time percentage was calculated by Equation 9:

$$t_{n}=t/T_{C}\times 100\%$$ (9)

Where t is the residence time that pump operates at a certain flow range, and $T_{C}$ is the total time of one cardiac cycle. The modulation method of the speed will be discussed below as two cases: in-phase and counter-phase conditions (May-Newman, 2023).

#### 3.1.1 In-phase condition

When the variable speed modulation is in-phase with the left ventricle (Modes 3, 4, 7, and 8 in Figure 4), the resulting characteristic loop is larger, maintaining a counterclockwise rotation. With the same baseline speed but varying speed fluctuations, an increase in speed variation (Mode 3 vs Mode 4, Mode 7 vs Mode 8) enlarges the characteristic loop. This indicates that when the blood pump operates in complete synchronization with the heart, its pumping capacity aligns with that of the left ventricle. As a result, the blood pump outputs higher pressure flows during high pumping capacity and lower pressure flows during low pumping capacity.

Under in-phase conditions, the blood pump’s flow distribution is wide, with prolonged periods at both minimum and maximum flow rates. Increasing the amplitude of speed fluctuations and the baseline speed expands the flow range within a cycle. This leads to more pronounced changes in the pump’s operating point, potentially enhancing the pulsatile effect of the blood flow, which could also increase the risk of hemolysis.

#### 3.1.2 Counter-phase condition

When the pump speed modulation is out of phase with the left ventricle (Modes 1, 2, 5, and 6 in Figure 4), the characteristic loop is smaller compared to the condition without pulsation, and the rotation direction shifts to clockwise (Modes 1, 2, and 5) or forms an “8”shape (Mode 6). Under counter-phase conditions, the direction of the pressure rise cannot be easily inferred from the flow direction. For the same baseline speed but different amplitudes, as the speed fluctuation increases (e.g., from Mode 6 to Mode 5), the reverse characteristic loop enlarges, shifting from an “8”shape to a fully clockwise loop. This suggests that when the blood pump operates entirely out of sync with the heart, a counterclockwise characteristic loop may form with low-speed pulsations. But as the speed fluctuations intensify, the reverse effect becomes more pronounced, indicating that the pumping capacity of the blood pump in this mode can exceed that of the left ventricle and a clockwise characteristic loop forms. The impact of speed variation on the working point across the current baseline speed surpasses the effect of the left ventricle’s natural pulsations.

In counter-phase conditions, the flow and pressure rise ranges are no longer evenly distributed across the four stages. For example, in Mode 1, with a baseline speed of 2,200 rpm and a modulation amplitude of 200 rpm, the flow range in the n0∼n0+A stage is from 94% to 3%, and the pressure rise range is from 100% to 90%. However, during the third stage, an increase in pressure rise occurs with little change in flow. In the fourth stage, a significant increase in flow occurs with little change in pressure rise. These stages correspond to a reduction in the left ventricle’s functional capacity, yet they do not result in the pressure or flow reductions in constant speed or in-phase modulation conditions.

The flow distribution graph of the blood pump reveals that under out-of-phase conditions, the flow distribution is narrow. With a baseline speed of 2000 rpm and modulation amplitudes of 200 rpm and 100 rpm, the pump remains in the mid-range flow for the longest duration. This is due to the characteristic loop exhibiting an “8”shape. At low-pressure rise points, there is a repeated fluctuation of flow decrease, increase, and decrease, causing the flow to remain near the mid-range. Consequently, in this condition, the high-risk area for blood damage is near the low-pressure rise point.

### 3.2 Hemolysis performance

**FIGURE 5:**
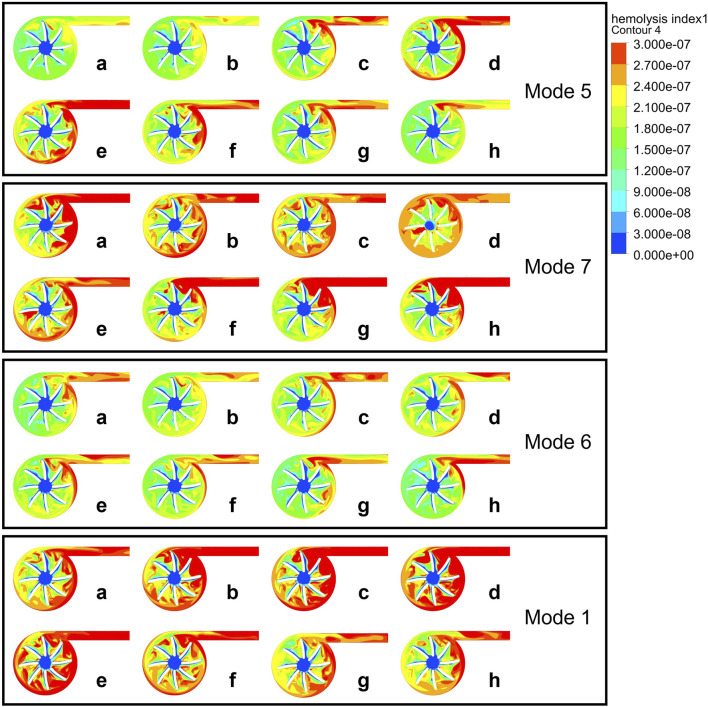
HI distribution contour maps at half the blade span height of the pump during a cardiac cycle in Mode 5, 7, 6, 1.

**FIGURE 6:**
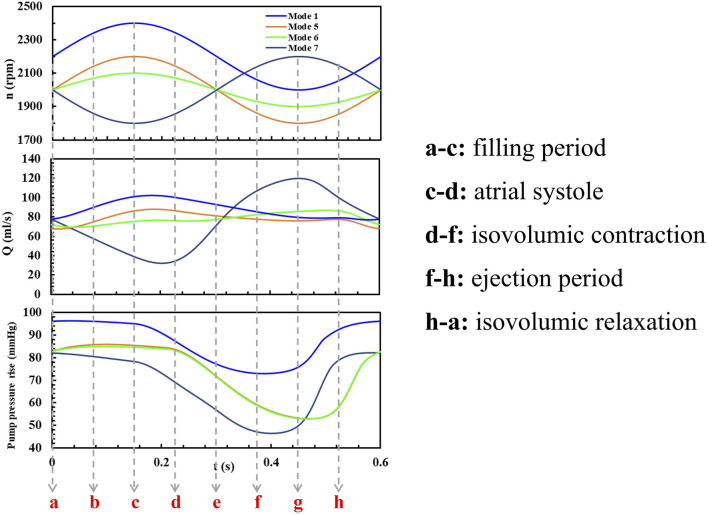
The pump speed, flow rate and pressure rise within a cardiac cycle in Mode 1, 5, 6, 7.

The influence of phase, baseline speed, and speed fluctuation amplitude on the hydrodynamic performance of the blood pump was evaluated using CFD simulations. As shown in Figures 4A, B, the characteristic curve of the blood pump exhibits a “8” pattern, with a point where both the flow rate and pressure rise are identical, but the trends differ. The appearance of the point indicates that the transient changes in the flow field within the pump are closely linked to the flow field formed in the previous time point, as well as to the varying conditions at the pump’s inlet and outlet. This is an issue not typically considered in the operation of a constant-speed blood pump. Therefore, it is necessary to further analyze the HI distributions within the pump (Fraser et al., 2012; Roberts et al., 2020; Yun et al., 2020). Figure 5 present the HI distribution within the pump over one cycle in Mode 1, Mode 5, Mode 6 and Mode 7, with the corresponding time points marked as Figure 6.

The left ventricle pressure remains stable at points a-d, after which the left ventricle begins to contract and work, with the pressure gradually increasing to point g and then gradually decreasing.

As shown in Figure 5, high HI is observed in the rotating region of the impeller blades and near the outlet. As the rotational speed and the coupling effect with the heart change, the distribution characteristics of HI change. Compared to Mode 5, Mode 7 changes the phase, Mode 6 changes the amplitude, and Mode 1 changes the baseline. During the period from n0 to n0+A (a-c), high HI gradually appears at the pump’s tongue and outlet in Mode 5. Due to the increase in left ventricular pressure starting from point d, and the severe deceleration of the blood pump speed at point e, there is a strong shear stress within the blood pump, which leads to the elevated hemolysis during this period.

According to Figure 4B, the characteristics of Mode 6 fit the steady-state characteristic curve most closely, indicating a smaller change in hemolysis within the blood pump in this operating condition. The point a and d of Mode 6 have almost the same pressure rise and flow rate, but the hemolysis at point *a* is still mainly concentrated at the outlet and the blades near the outlet, while at point d, hemolysis begins to occur at the trailing edge and volute.

Mode 7 has a relatively high possibility of hemolysis throughout a cardiac cycle, with its high HI distribution in all blade rows. Its in-phase adjustment method can cause irregular suction on the ventricle, thereby changing the inlet conditions and potentially increasing the risk of hemolysis.

Mode 1 has a baseline speed modulation at 2200 rpm, with an average speed higher than the other speed modulation methods, leading to higher shear stress. According to the research of Chen et al. (2016), non-physiological high shear stress can lead to platelet activation and hemolysis. Areas inside the blood pump with high shear stress are potential danger zones for blood damage, which is consistent with the high hemolysis risk we observed in Mode 1. Within a cardiac cycle, the hemolysis distribution in Mode 1 varies in a consistent with Mode 5, indicating that under similar inlet and outlet conditions, flow field characteristics inside the blood pump is similar.

**FIGURE 7:**
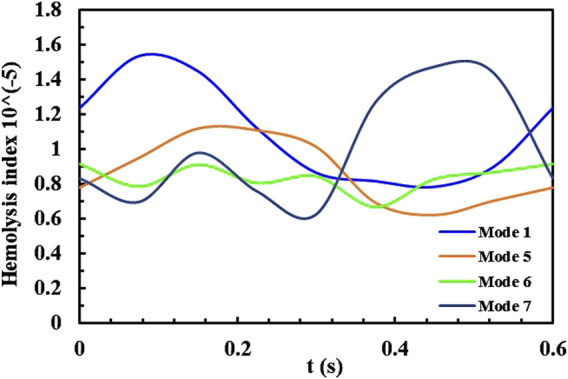
The average HI during a cardiac cycle in Mode 5, 7, 6, 1.

Figure 7 shows the plotted curve of the mass-weighted average HI value at the pump outlet under four speed modulation conditions. There is a consistent trend between the average HI value and pump speed. Mode 1 causes the most significant fluctuation in HI under the counter-phase speed modulation mode, reaching a maximum HI value when the speed reaches its peak. Other reverse-phase speed modulation modes maintain relatively stable pump operating characteristics, resulting in less fluctuation in HI throughout a cardiac cycle. Mode 7, as a in-phase speed modulation mode, also reaches a maximum HI value when the speed is at its peak, but at the lowest speed, HI will also experience a slight increase. The average hemolysis value for all modulation modes is $9.49^{-6}$ , with Mode 6having the lowest average HI and Mode 1 having the highest average HI, consistent with the analysis results above.

## 4 Discussion

This study aims to provide a rapid estimation of the performance of a variable speed blood pump under coupled working conditions with the cardiovascular system using CFD and LPM methods. Specifically, it investigates the impact of two types of sinusoidal speed variations of the blood pump: one in-phase and one out-of-phase with the left ventricular pressure pulsation waveform. By setting baseline speeds at 2,200 rpm and 2000 rpm, with speed fluctuations of 200 rpm and 100 rpm respectively, the study explores eight different support modes to simulate the blood pump’s support scenarios in heart failure patients.

Hemolysis is a key performance indicator for blood pumps. Many researchers have adopted numerical hemolysis assessment using CFD methods in the design optimization of pumps (Wu et al., 2006; Wiegmann et al., 2018). It serves as a good supplement to *in vivo* and *in vitro* hemolysis tests. Although there have been numerous studies on the effects of constant rotational speed on blood pumps, research on the impact of variable speed modulation on centrifugal pump performance is still insufficient. This study employs CFD and 0D modeling techniques to conduct a comprehensive comparative study of the numerical hemolysis performance of a centrifugal pump under constant speed and eight different speed modulation curves. The findings have significant reference value for the actual clinical speed operation of blood pumps.

According to Figure 5, the main changes in HI occur at the blade trailing edge, volute tongue, and outlet duct. The increase in HI at the blade trailing edge and volute tongue is mainly due to the increase in shear stress, while the outlet duct has lower shear stress but a significant increase in residence time leads to an increase in HI. The settings of the blood pump’s phase, baseline speed, and speed fluctuation amplitude all affect the hemolytic performance of the blood pump. Mode 6, compared to Mode 5, reduces the amplitude, making it have a smaller risk of hemolysis, but it results in a smaller blood flow pulsation as shown in Figure 3E. Mode 1, compared to Mode 5, has a higher baseline speed, and the increase in speed leads to an increased risk of hemolysis (Jahren et al., 2014). Mode 7, as an in-phase speed modulation method, works in synchronization with the left ventricle and produces a more noticeable pulsatile flow. However, due to the fluid inertia within the blood pump blades, large shear stress can occur here when the rotor suddenly decelerates or accelerates, leading to an increased risk of hemolysis.

**FIGURE 8:**
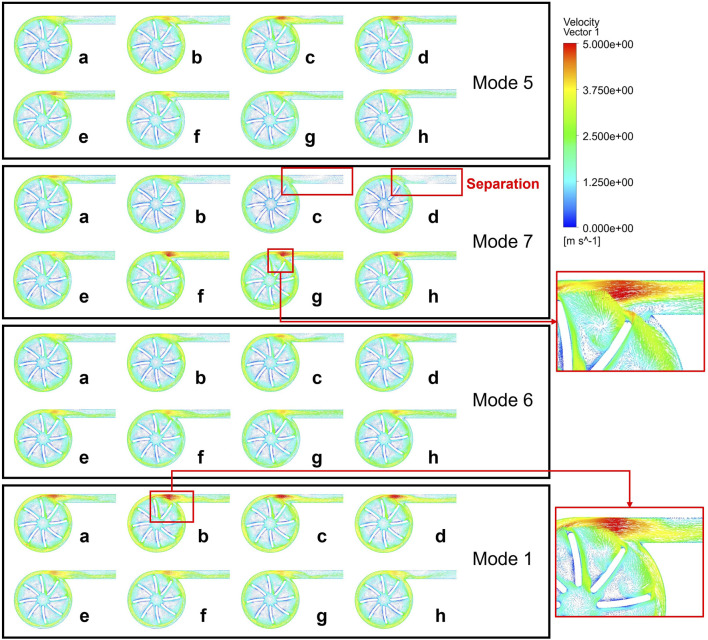
Velocity vector distributions during a cardiac cycle in Mode 5, 7, 6, 1.

As shown in Figure 8, to further explore the relationship between the flow structure inside the blood pump and hemolysis, we conducted a comparative analysis of the velocity vector distributions inside the blood pump under these modulations (Yang et al., 2015; Wu et al., 2010; Chassagne et al., 2023). The distribution indicates that when the left ventricular pressure is relatively stable (a-d), Mode 7 shows a significant large separation zone at the outlet due to the decrease in rotational speed, leading to blockage at the outlet and a reduced flow rate, which is corresponding to the complex hemolysis characteristics at the outlet in the hemolysis distribution diagram. With the synchronous increase in pump speed and left ventricular pressure, vortices appear in the blade area, the flow rate increases, and a high-speed zone appears at the tongue of the volute, but the flow distribution inside the outlet conduit is relatively uniform. During point a to d, both Mode 5 and Mode 6 have high-speed zones of the tongue and large separation at the outlet, caused by an increase in pump speed but insufficient inlet pressure, preventing the increase in flow. As the rotational speed gradually decreased and the inlet pressure gradually increased, the turbulence within the blade passage was significantly enhanced, but the separation at the outlet improved. The flow field distribution and variation trend of Mode 1 are similar to those of Mode 5 and Mode 6, but due to its higher reference velocity, the internal flow structure is more complex, the separation zone is larger, and thus causes more severe hemolysis.

The change in pump speed resulted in the formation of more vortices within and at the outlet and blade passage, and a more complex vortex structure was observed at the time points when hemolysis was more severe, flow separations are noticed in the domains of impeller and outlet tube. As mentioned, these irregular flow patterns may aggravate the hemolysis. Due to the fact that the work of a centrifugal pump mainly comes from centrifugal force, the design of a centrifugal blood pump typically requires a certain volume to ensure a significant radius difference between the leading and trailing edges of the impeller. At high rotational speeds, the tangential velocity at the trailing edge is further increased. During the acceleration process, the velocity gradient between the blade trailing edge and the centrifugal pump volute increases, leading to an increase in shear stress and thus an increase in the possibility of hemolysis. The quality of the blood flow at the outlet of the blood pump is closely related to the quality of the blood supply. Blood with larger separation or vortex structures flowing into the aorta through the catheter may lead to imbalanced blood flow supply to different arteries or hemolysis caused by impacts. Moreover, the excessive suction of blood flow into the left ventricle caused by blockage at the outlet can easily lead to ventricular collapse or direct suction into the myocardium. Therefore, conducting effective simulations of variable-speed blood pumps before actual variable-speed blood pump configurations is of utmost importance.

Understanding how different operational modes affect blood pump performance and blood damage potential is crucial for optimizing LVAD therapy. The findings from this study could lead to improved LVAD designs and operational protocols, ultimately enhancing patient outcomes by providing more tailored and effective mechanical circulatory support. This research also highlights the importance of personalized medicine in managing advanced heart failure, ensuring that each patient receives the most appropriate and beneficial therapy based on their unique physiological characteristics. The focus on variable speed operation underscores the potential for more natural and effective support strategies, moving beyond the limitations of constant speed pumps.

This study still has some limitations. The research is based on a 0-dimensional human circulatory model, which provides boundary conditions for CFD simulations that have a certain degree of physiological significance and have been recognized for their accuracy by various scholars. However, in clinical practice, factors such as baroreflex effects and variations in left ventricular elasticity can influence physiological states, which are often not static. Therefore, in the future, we might consider adding a speed input function to the blood pump’s speed controller to achieve active control. Additionally, since the results indicate that there are different characteristic curves for the eight speed modulation methods, but these are only implemented under conditions that are in sync with the cardiac cycle, future research could explore hemolysis assessment when the cardiac cycle is independent of the blood pump speed modulation cycle.

## 5 Conclusion

This study demonstrates that the hydrodynamic performance and hemolytic risk of rotary blood pumps are profoundly shaped by the interplay of phase, baseline speed, and speed fluctuation amplitude. Through computational modeling and dynamic cardiovascular coupling, we have identified distinct impacts on flow patterns and blood damage potential. The results indicate that both the in-phase and counter-phase speed modulations offer specific benefits and drawbacks, with varying degrees of impact on hemolysis, especially in areas of high shear stress within the pump. The study highlights the importance of optimizing pump speed amplitudes, baseline speed and phase differences to align more closely with the patient’s physiological needs. In-phase modulation tends to enhance pulsatile flow but may increase hemolysis under certain conditions, while counter-phase modulation appears to provide more stable flow characteristics, albeit with a more complex flow structure that can still pose risks.

The analysis of pressure-flow hysteresis curves further highlights the complexity of heart-pump interactions: counter-phase modulation generates “8”loops, indicative of transient flow reversal that mitigates hemolysis, while in-phase conditions produce clockwise loops (Mode 7) and pulsatility-driven shear stress in the volute tongue.

Flow distribution statistics reveal that under counter-phase modulation, the pump operates within the mid-flow range for most of cardiac cycle, significantly reducing exposure to high-shear zones. These findings collectively emphasize that timing precision in speed modulation—rather than waveform shape alone—determines the balance between pulsatility and blood compatibility.

This study demonstrates that variable-speed modulation is not merely an engineering optimization problem but a physiological adaptation strategy. By dynamically adjusting pump speed to match cardiac cycle phases, VADs can preserve the endothelial cell response to pulsatile shear stress—a key mechanism for vascular homeostasis. Furthermore, minimizing hemolysis through speed control aligns with the physiological need to maintain erythrocyte integrity, thereby preventing hemoglobin-induced nephrotoxicity and oxidative stress. These insights advance the translational goal of designing VADs that not only support circulation but also actively promote cardiovascular system resilience. Future work should explore the clinical implications of these findings, potentially integrating real-time physiological feedback to optimize pump performance dynamically. Moreover, expanding the simulation to account for more complex patient-specific factors such as baroreflex and variable ventricular elasticity could further refine the use of variable-speed pumps in heart failure management. The ultimate goal is to enhance mechanical circulatory support, making it safer and more effective for patients with heart failure.

## Data Availability

The original contributions presented in the study are included in the article/supplementary material, further inquiries can be directed to the corresponding author.
